# Effect of Sea Buckthorn on Plasma Glucose in Individuals with Impaired Glucose Regulation: A Two-Stage Randomized Crossover Intervention Study

**DOI:** 10.3390/foods10040804

**Published:** 2021-04-08

**Authors:** Zhongxia Ren, Huiting Gong, Ai Zhao, Jian Zhang, Chenlu Yang, Peiyu Wang, Yumei Zhang

**Affiliations:** 1 Department of Nutrition and Food Hygiene, School of Public Health, Peking University Health Science Center, Beijing 100191, China; renzhongxia@bjmu.edu.cn (Z.R.); huitinggong@163.com (H.G.); zhangjian92@pku.edu.cn (J.Z.); yangchenluwork@126.com (C.Y.); 2 Vanke School of Public Health, Tsinghua University, Beijing 100091, China; aizhao18@tsinghua.edu.cn; 3 Department of Social Science and Health Education, School of Public Health, Peking University Health Science Center, Beijing 100191, China; wpeiyu@bjmu.edu.cn

**Keywords:** sea buckthorn, impaired glucose regulation, plasma glucose, randomized crossover intervention study

## Abstract

Sea buckthorn (SB) has been indicated to have hypoglycemic potential, but its effects on glucose in people with impaired glucose regulation (IGR) are still unclear. This work presents a randomized, double-blinded, two-way crossover study. A total of 38 subjects with IGR completed the intervention of consuming sea buckthorn fruit puree (SBFP, 90 mL/day, five weeks), washing out (four weeks), and then consuming placebo (90 mL/day, five weeks) or in reverse order. In our methodology, a unified questionnaire was used to gather information on physical activity and dietary intakes, and physical examinations were performed to measure blood pressure, height, and weight. Fasting blood samples were collected to detect the fasting plasma glucose (FPG) and glycated serum protein (GSP). To calculate the area under the curve of 2 h postprandial plasma glucose (2 h PG-AUC), blood samples at t = 30, 60, and 120 min were also collected and analyzed. Effects of the intervention were evaluated by paired-sample Wilcoxon test and mixed model analyses. Our results show that the FPG in subjects with IGR decreased by a median reduction of 0.14 mmol/L after five weeks’ consumption of SBFP, but increased by a median of 0.07 mmol/L after placebo intervention, and the comparison of these two interventions was statistically significant (*p* = 0.045). During the wash-out period, a similar difference was observed as the FPG decreased in the group that received SBFP intervention first, but increased in another group (*p* = 0.043). Both SBFP and placebo significantly raised GSP during the intervention period, but lowered it in the wash-out period (*p* < 0.05), while no significant difference was found between the two interventions. The 2 h PG-AUC remained relatively stable throughout the study. Our results indicated that consumption of SBFP for five weeks showed a slight downward trend on FPG in subjects with IGR.

## 1. Introduction

Prevention of type 2 diabetes mellitus (T2DM) has become a major health challenge given its high global prevalence, which has reached 8.5%, according to the latest report on diabetes published by the World Health Organization (WHO) in 2016 [1]. Impaired glucose regulation (IGR), also known as prediabetes, including impaired glucose tolerance (IGT) and/or impaired fasting glycemia (IFG), are insidious intermediate conditions before the occurrence of T2DM [2]. Considering China’s high prevalence of IGR, which has reached 35.7% (95% confidence interval: 34.1–37.4%) in 2013 and been maintained at 35.2% (95% confidence interval: 33.5–37.0%) in 2017 [3,4], it is of practical significance in preventing the conversion of prediabetes to T2DM.

Dietary and/or another lifestyle intervention has been listed as effective strategies in the international guidelines on the management of prediabetes [5,6,7]. As a kind of food rich in bioactive compounds, berries are considered “promising functional fruits” and have been widely studied in recent years [8,9,10]. A systematic review and meta-analysis of prospective cohort studies showed that consumption of berries was associated with an 18% reduced risk of T2DM [11]. The incorporation of berries or berries-derived byproducts into high-carbohydrate (HCD) and high-fat (HFD) diets, has also been found to contribute to the reversion/reduction of the HCD/HFD-induced alterations in glucose metabolism-related pathways and inflammation in diabetic subjects [8].

*Hippophae rhamnoides* L., commonly referred to as sea buckthorn (SB), belongs to the family Elaeagnaceae and genus *Hippophae* [12]. At present, increasing attention on the possible positive effects of SB berries for glycemic control was also noted. Several animal studies have shown positive effects of SB protein/fruit oil extract on reducing blood glucose, as well as alleviating insulin resistance [13,14,15]. In human trials, for example, compared with strawberry, SB decreased and delayed insulin response and improved glycemic profile following a sucrose-containing berry meal [16]. In another randomized study, a daily dose of 100 g fresh SB reduced fasting plasma glucose (FPG) after intervention for 33–35 days, although the proportion of glycated hemoglobin A1c (HbA1c) was increased contrarily [9]. Elsewhere, 40 g of dried SB together with yogurt (200 g) and glucose (50 g) has been suggested to stabilize postprandial hyperglycemia and suppress peak insulin response [17].

As reviewed by Padwad et al., all parts of SB have been found to be a rich source of bioactive substances, such as fat-soluble vitamins (A, E, and K) and flavonoids (quercetin, kaempferol, isorhamnetin, myricetin) [18]. The flavonoids, in particular, have been indicated to potentially exhibit hypoglycemic activities through reducing glucose absorption, enhancing insulin secretion and sensitivity, regulating hepatic glucose output, among others [19,20]. In addition, SB may also play a role in regulating neuroendocrine, immune, antioxidant, and anti-inflammatory functions [21,22,23], which might contribute to the control of blood glucose.

Although the studies above showed positive effects of SB on glycemic control, it should be noted that most of these studies were conducted in overweight and/or obese subjects [9,16]. Furthermore, previous studies have been carried out mainly in Europe, using European SB berries as the intervention [16,17]. According to records, SB has a long history of cultivation and edible or medicinal use in China [24]. In addition, five of the nine subspecies of SB are distributed in China, which suggests great research potential [25]. Recently, a previous double-blind, randomized, placebo-controlled trial conducted by our team showed that the high-sensitivity C-reactive protein concentration was decreased in the SB group in patients with hypercholesterolemia, indicating an anti-inflammatory effect [26]. However, the possible benefits of SB in IGR subjects are still unknown.

Thus, this study aimed to assess the effects of SB grown in China on glycemia indicators, including FPG, postprandial plasma glucose (PPG) and glycated serum protein (GSP) in subjects with IGR.

## 2. Materials and Methods

### 2.1. Study Design

This was a two-stage, randomized crossover and double-blind intervention study. It was implemented in accordance with the Declaration of Helsinki and approved by the Medical Ethics Research Board of Peking University (No. IRB00001052-18054). It also has been registered at the Chinese Clinical Trial Registry (No. ChiCTR1800019450).

**Figure 1:**
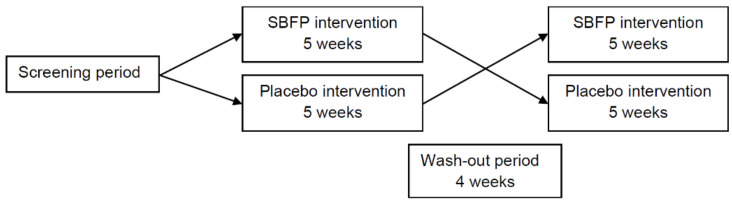
Two-stage and randomized crossover design of this study. SBFP, sea-buckthorn fruit puree.

After the screening visit, subjects were randomly divided into two groups (AB and BA). The intervention regimen of group AB consisted of drinking sea-buckthorn fruit puree (SBFP) for five weeks, washing out for four weeks, and then drinking placebo for another five weeks, while the order of group BA was reversed (Figure 1). The total consumption of SBFP and placebo was 90 mL/day, which should be taken with three meals. Volunteers were asked to maintain their habitual lifestyle throughout the study period. A total of four follow-up visits were conducted on day 0 (before the first phase of intervention), day 35 (after the first phase of intervention), day 63 (before the second phase of intervention), and day 98 (after the second phase of intervention). Based on a previous study, the elimination half-life of isorhamnetin, one of the most stable SB flavonoids, was around 118.3 h in rats [27]. Thus, to ensure five times the elimination half-life (591.5 h ≈ 25 days) and individual metabolic differences, 28 days was taken as the wash-out period.

### 2.2. Participants

**Figure 2:**
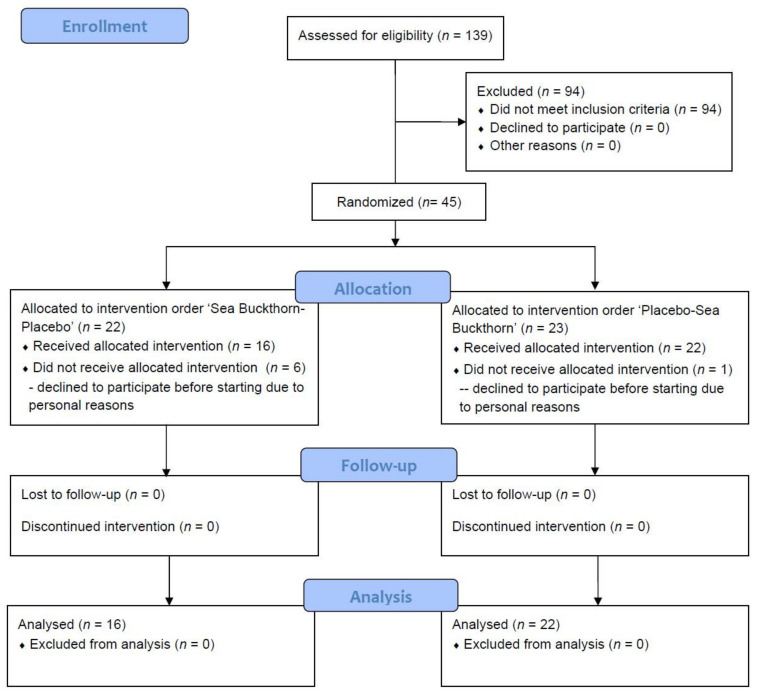
Flow diagram of participation at baseline and follow-up.

A total of 139 subjects were enrolled in the study—of which, 45 were randomly grouped, and 38 completed the study (Figure 2).

Volunteers with IGR were recruited through new media and recruitment notices in Beijing, China. All participants were informed of the details of the study and gave written consent to participate. At the screening visit, fasting blood samples were collected, and screening questionnaires were used to assess each volunteer’s health status. Inclusion criteria comprise patients aged 50–70 years with IGR (6.1 mmol/L ≤ FPG < 7.0 mmol/L and/or 5.6% ≤ HbA1c ≤ 6.5%). Exclusion criteria comprise diabetes or use of hypoglycemic drugs, clinical interfering conditions (thyroid, kidney, liver, blood, immune-related diseases, infectious diseases, gastric ulcer), participation in other intervention studies or use of hypoglycemic or sea buckthorn-related dietary supplements, irregular diet, mental illness, and memory impairment.

### 2.3. Test Products

The test products included SBFP and placebo produced by the Highland Holy Fruit Company Limited. The raw SB (*Hippophae rhamnoides* L. *subsp. Sinensis*) was provided by the Sea Buckthorn Development and Management Center of the Ministry of Water Resources (Ordos, China), which was frozen immediately after harvesting in October 2017. During the production process, the seeds were removed, and the peel and pulp were preserved and squeezed into puree. After low-temperature aseptic treatment, the puree was filled into brown glass bottles with a volume of 30 mL/bottle. The contents of active components in SBFP were detected by China Agricultural University (Table 1). The analysis of sugars, fruit acids, and ascorbic acid by gas chromatography (GC) and flavonol glycosides by high-performance liquid chromatography (HPLC) were performed as previously described [28]. The fiber was determined by the enzymatic-gravimetric method, according to the Chinese Standard GB 5009.88-2014 [29].

Formulation of the placebo was consigned to China Agricultural University for design, according to the study of Eccleston et al. [30], while the production and filling were consigned to Highland Holy Fruit Company Limited, using the same specification as the SBFP. The appearance and taste of the two test products were near-identical, and their formulae are shown in Table 2. In order to assess the compliance, participants were asked to return their empty bottles at each follow-up visit.

### 2.4. Measurements

#### 2.4.1. Lifestyle Questionnaire

At each visit, a questionnaire survey was conducted to obtain the basic information, living habits, physical activity level, and dietary intake during the study period. Data on weekly physical activity were collected using the International Physical Activity Questionnaire (long version), and the metabolic equivalent (MET) was calculated. Daily sitting time and sleeping time (min/day) were also recorded. Information about food consumption in the past 24 h before every visit was obtained by a one-off 24 h dietary recall with the help of standard bowls, plates, spoons, and reference picture books. Several main nutrients, including energy, protein, total fat, carbohydrate, fiber, and cholesterol, were then calculated based on the Chinese Food Composition Table (second edition) [31].

#### 2.4.2. Physical Examination

Physical examination consisting of blood pressure, height, and weight were taken with standard protocols. For patients in a calm state, the systemic blood pressure (SBP) and diastolic blood pressure (DBP) at the brachial artery of the right upper arm were measured by Omron HEM-7124 electronic sphygmomanometer twice, and the mean value was calculated. Body mass index (BMI) was calculated using the standard formula: BMI = weight (kg)/height (m^2^).

#### 2.4.3. Blood Samples

At each visit, blood samples were collected from the antecubital vein. Subjects were reminded to fast for 8–10 h before the visit to collect the fasting venous blood samples, with 2 mL in the ethylene diamine tetraacetic acid (EDTA) anticoagulant tube and 4 mL in the non-anticoagulant tube. Then a standard meal was given, and blood samples were also drawn at 30, 60, and 120 min after the meal (4 mL in the non-anticoagulant tube). The standard meal was a fixed brand of white bread (75 g, energy 1345 kJ/100 g, carbohydrate 49.9 g/100 g, protein 9.0 g/100 g, fat 9.3 g/100 g) and a bag of pure milk (227 mL, energy 261 kJ/100 mL, carbohydrate 4.5 g/100 mL, protein 3.0 g/100 mL, fat 3.6 g/100 mL).

All the blood samples were stored in a 4 °C refrigerator and sent for examination within 2 h. The blood samples in the non-anticoagulant tube were centrifuged, and its serum was used to detect plasma glucose (PG) by the glucose oxidase methods. The GSP was measured by the ketamine oxidase method, respectively, using the whole blood sample in the EDTA anticoagulant tube. The above indicators were all tested by a qualified laboratory (Lawke Health Laboratory, Beijing, China), using Roche automated production analyzer (Cobas C501; Roche Diagnostics Co., Ltd., Rotkreuz, Switzerland).

The area under the curve of 2 h postprandial PG (2 h PG-AUC) was calculated using the following trapezoidal area formula:

(1) $$2h\;\mathrm{PGAUC}=\frac{(\mathrm{FPG}+\mathrm{PG}\;30\;\min)\times 0.5}{2}+\frac{(\mathrm{PG}\;30\;\min+\mathrm{PG}\;60\;\min)\times 0.5}{2}+\frac{(\mathrm{PG}\;60\;\min+\mathrm{PG}\;120\;\min)\times 0.5}{2}$$

### 2.5. Statistical Analyses

R version 3.6.3 was used for data analysis. The intention-to-treat (ITT) analysis was applied. Continuous variables were presented as median (interquartile range, IQR), and categorical variables were expressed as frequency (percentage). To verify the basic assumptions of crossover design, the carryover effect was evaluated using the independent-sample Wilcoxon test, which compared the sum of the blood glucose-related indices (FPG, 2 h PG-AUC, GSP) at the end of both intervention periods between group AB and BA. The differences before and after the intervention of SBFP or placebo were evaluated with paired-sample Wilcoxon test. The differences in physical activity and dietary intakes during the whole study period (among the four follow-up visits) were compared by the Friedman rank sum test. As for glycemic markers and physical examination indicators, mixed-model analyses were conducted. The intervention, order (group), and period were treated as fixed effects and individual as a random effect. Changes of the indices before and after the wash-out period were determined by the two-factor repeated measurement ANOVA. All analyses assumed a two-sided test of hypothesis, and the statistical significance was indicated by *p* < 0.05.

## 3. Results

A total of 45 volunteers were randomly grouped for the study—seven of them voluntarily withdrew prior to the start, with the remaining 38 participating through to the study’s conclusion. No adverse effects were observed during the study period.

Baseline characteristics can be seen in Table 3. The average age of the 38 subjects who completed the whole intervention was 59.1 ± 4.8, and 42.1% were older than age 60. About 80% of the participants were female (*n* = 30), and 28 of the 30 were postmenopausal. The carryover effects of all the blood glucose-related indices (FPG, 2 h PG-AUC, GSP) were not significant (*p* > 0.05).

### 3.1. Intervention Period

#### 3.1.1. Lifestyles

All patients’ physical activity metrics, including METs, sitting time, and sleeping time, did not change significantly before and after the interventions (Table 4). For dietary intakes, an increase of carbohydrate was observed during the intervention period of SBFP, while the difference was not significant throughout the whole study period.

#### 3.1.2. Glycemic Markers

The FPG in subjects with IGR decreased by a median reduction of 0.14 mmol/L after five weeks of consumption of SBFP, but increased by a median of 0.07 mmol/L after placebo intervention. After adjustment, the difference in the two interventions was significant (*p* = 0.045), according to the mixed model analyses. SBFP or placebo resulted in a median increase of 0.40 or 0.23 h mmol/L on 2 h PG-AUC, respectively, while no significant difference was observed between two interventions (*p* = 0.871), as well as the PG at 30, 60, and 120 min. Both SBFP and placebo interventions raised GSP significantly (*p* < 0.05), while the mixed-model analyses did not show a significant difference between the two interventions (Table 5).

#### 3.1.3. Physical Examination Indicators

The patients’ BMI and SBP did not change significantly throughout the study. In addition, although both SBFP and placebo lowered DBP significantly (*p* < 0.05), the difference between the two interventions was not significant (Table 6).

### 3.2. Wash-Out Period

During the wash-out period, the FPG decreased by a median reduction of 0.29 mmol/L in group AB (received SBFP at the first intervention stage), but increased by a median of 0.10 mmol/L in group BA (received placebo at the first intervention stage). The effect between the two groups was significant (*p* = 0.043). The GSP decreased significantly in both groups, but the difference between the two groups was not significant, nor was 2 h PG-AUC (Table 7).

## 4. Discussion

According to the screening data, the detection rate of IGR was 32.4% (45/139), which was close to the prevalence of 35.2% reported in a nationally representative cross-sectional survey in China in 2017 [4]. Considering that IGR contributes to a high risk of type 2 diabetes and other metabolic diseases [2,32,33], it is crucial to investigate more effective prevention strategies [34,35]. The positive effect of functional foods rich in bioactive ingredients on preventing and managing chronic diseases, for example, is commonly accepted [7,36]. In the present study, consumption of 90 mL SBFP for 35 days led to a tendency for FPG to decrease in people with IGR, but did not affect the PPG or GSP.

To date, evidence of the ability of SB to alter FPG is insufficient and mainly produced by animal trials. Consistent with the positive effect found in this study, Lehtonen et al. reported that there was a small, but significant, decrease in FPG (−0.1 mmol/L; *p* = 0.002) after the inclusion of a certain amount of air-dried SB (equivalent to 100 g/day fresh berries) for 33–35 days in the diet of 110 overweight and obese women [9]. In a recent animal trial, after a four-week treatment period (100–200 mg/kg/day), the FPG of diabetic mice was significantly reduced in the SB seed protein (SSP) and SB polysaccharide (SPO) group compared with the module control group, whereas SB procyanidins (SPR) showed no effect [14]. Administration of flavonoids from sea buckthorn pomace (SBP) for four weeks also resulted in significantly hypoglycemic effects in ICR mice with alloxan-induced diabetes, even in the low dose group (3.0 mg/kg/day) [37]. In the study by Gao et al., it was suggested that a middle or high dose of sea buckthorn fruit oil (SBFO, 200 or 300 mg/kg/day) could lower the FPG at a rate of 10.47% and 13.79% in T2DM SD rats, respectively [13]. In addition, the continuous intervention of methanolic extract from *Hippophae salicifolia* D.Don (a species of SB) leaves at 200 or 400 mg/kg for 45 days both exhibited significant reduction (22% and 39%, respectively) in FPG compared to the diabetic control rats [38].

Prior research suggested that SB and its extracts might have a hypoglycemic effect. However, the mechanism of this effect was unclear. Protein included in SB is considered to be a high-quality resource of essential and semi-essential amino acids [14]. A study by Yuan et al. confirmed an increase of hypoglycemic-related beneficial bacteria (*Bifidobacterium* and *Lactobacillus*) in diabetic mice with consumption of SSP, which could be due to the higher concentrations of short-chain fatty acids and lower pH produced by the metabolism of SSP [39]. An in vitro experiment conducted in insulin-resistant HepG2 cells revealed that the SBFO extract rich in palmitoleic acid (POA) could increase the expression of glucose transporter type 4 through the activation of the PI3K/Akt signaling pathway [2]. In addition to the macronutrients mentioned above, evidence accumulated over the years indicates that biologically active substances rich in SB may also play an important role, especially polyphenols. The mechanisms involved in relevant studies, including the inhibition of glucose absorption, stimulation of insulin secretion and activation of insulin receptors, modulation of hepatic glucose output, etc. [7,19,20]. Based on these studies, abundant unsaturated fatty acids (e.g., POA) and flavonoids (especially Isorhamnetin and Quercetin, see Table 1) in the SB we used in this study may explain its positive role in hypoglycemic.

In line with expectations, the intervention of SBFP at five weeks showed a positive effect on FPG. However, during the wash-out period, subjects who received SBFP also had a continued decline of FPG, which might suggest long-term effects. Since the statistic carryover effects were not significant in our study, the wash-out period set in our study was considered reasonable. In future studies, the intervention can be extended appropriately, and the long-term effects of SB need to be examined.

When it comes to PPG, the results of previous studies were not completely consistent. The study by Gao et al. showed that four weeks’ treatment of SBFO extracts improved the oral glucose tolerance test (OGTT) in T2DM SD rats in a dose-dependent manner [13]. A study based on ten healthy volunteers consuming one control meal and three experimental meals with dried and crushed whole SB berries, supercritical fluid (SF)-carbon dioxide (CO2)-extracted oil-free berries, and ethanol-extracted SF-CO2-extraction residue, respectively, suggested that meal with dried and crushed whole berries stabilize postprandial hyperglycemia [17]. However, in the study by Mortensen et al., no difference between control and SB was observed for PPG following a sucrose-containing berry meal [16]. It needs to be noted that, distinct from the above studies, we measured PPG after a standard meal without SBFP, so the effect we detected was not immediate, and further studies are still needed.

To date, there are few studies concerning the effect of SB on HbA1c or GSP, which reflects the average PG level over three months or 2–3 weeks, respectively. Nemes-Nagy et al. treated 30 type 1 diabetic children with a dietary supplement containing blueberry and SB for two months, and levels of HbA1c were significantly lowered in this group [40]. However, it is hard to distinguish whether the effects are a consequence of SB or blueberry treatment. Regretfully, the intervention of our study was not long enough to reflect the status of blood glucose in the last three months. But to the best of our knowledge, this is the first study to report the effect of SB on GSP. Reflecting a short-term glycemic regulation, GSP increased in the intervention period, but decreased in the wash-out period, and it seems to have no significant relevance to different interventions.

Previous studies indicated that berries may help improve appetite control, due to their fiber content, which in turn facilitates the control of weight [41]. An increased desire for something sweet was observed in the study by Mortensen et al. after administering a test meal containing added sucrose and SB [16]. In the present study, no differences for SB or placebo were observed for any nutritional intakes. However, whether the one-off 24 h food recall represents relevant nutrient intake is unclear. The effects of SB on appetite or dietary intakes, and its association with PG, need further study.

The strength of this study lies in the crossover design and the zero-dropout rate after the start of this study. To our knowledge, this is the first study to use SBFP for patients with IGR, and we evaluated relatively comprehensive glycemic markers, including FPG, 2 h PG-AUC and GSP. However, there are some limitations that should be considered. The distribution of genders was not balanced—although most women in our study had reached menopause, the differences in biology and physiology between men and women cannot be ignored. Furthermore, we did not provide long-term follow-up to detect the HbA1c and the progression from IGR to diabetes or normal glucose regulation, highlighting potential directions for future research.

## 5. Conclusions

In the present study, the consumption of SBFP for five weeks showed a slight downward trend on FPG in subjects with IGR. The potential of SB as a candidate berries for hypoglycemic deserves further investigation with a long-term follow-up.

## Figures and Tables

**Table 1:** Contents of active components in sea buckthorn fruit puree.

Component	Concentration (g/100 mL)
Total sugars (sugars, sugar alcohols, and derivatives)	1.60
Glucose	1.14
Fructose	0.17
Malic acid	3.15
Quinic acid	1.10
Ascorbic acid	0.26
Total flavonoids	93.02
Isorhamnetin-3-*O*-sophoroside-7-*O*-rhamnoside	10.86
Isorhamnetin-3-*O*-glucoside-7-*O*-rhamnoside	34.94
Isorhamnetin-3-*O*-rutinoside	26.91
Isorhamnetin-3-*O*-glucoside	6.70
Quercetin-3-*O*-sophoroside-7-*O*-rhamnoside	3.76
Quercetin-3-*O*-rutinoside	6.26
Quercetin-3-*O*-glucoside	3.59
Fiber	30.10 g/100 g (dried fruit)

**Table 2:** Formulae of sea-buckthorn fruit puree (SBFP) and placebo.

Formula	SBFP (%)	Placebo (%)
Sea-buckthorn fruit	95.7	-
Sweeteners (aspartame, acesulfame potassium)	2.9	1.2
Thickeners (guar gum, locust bean gum)	0.9	0.9
Acidity regulator (5 M NaOH)	0.6	-
Water	-	90.3
Malic acid	-	3.5
Fructose	-	2.7
Bread crumbs	-	1.4
Sea-buckthorn spices and colorants (lemon yellow, cochineal red, medicinal charcoal)	-	0.02

**Table 3:** Baseline characteristics of the participants.

Characteristics	Values ^a^
Gender, *n* (%)	
Male	8 (21.1)
Female	30 (78.9)
Age, *n* (%)	
50–60 years	22 (57.9)
61–70 years	16 (42.1)
Glycemic markers	
FPG, mmol/L	5.78 (0.85)
PG 30 min, mmol/L	6.55 (1.47)
PG 60 min, mmol/L	6.70 (2.18)
PG 120 min, mmol/L	6.24 (1.62)
2 h PG-AUC, h mmol/L	13.11 (2.89)
HbA1c, %	5.90 (0.38)
GSP, mmol/L	2.20 (0.20)
Physical examination indicators	
SBP, mmHg	120.50 (20.50)
DBP, mmHg	79.00 (12.25)
BMI, kg/m^2^	25.12 (4.22)
Physical activity	
METs (min/week)	3298.00 (4016.75)
Sitting time (min/day)	184.29 (135.00)
Sleeping time (min/day)	420.00 (60.00)

^a^ Values are presented as frequency (percentage) or median (interquartile range).

**Table 4:** Changes in physical activity and dietary intakes during the study period.

	SBFP (*n* = 38)				Placebo (*n* = 38)				*p* ^b^
	Before	After	Change	*p* ^a^	Before	After	Change	*p* ^a^	
Physical activity (min)									
METs/week	3298.00 (2649.00)	2893.50 (3339.00)	−579.25 (2999.88)	0.507	3797.75 (4171.75)	4120.50 (3582.63)	−237.00 (2372.38)	0.400	0.774
Sitting time/day	205.71 (196.07)	240.00 (118.93)	2.14 (97.5)	0.638	231.43 (106.07)	240.00 (207.86)	8.57 (97.5)	0.056	0.249
Sleeping time/day	420.00 (62.14)	420.00 (112.50)	0.00 (69.64)	0.533	420.00 (90.00)	428.57 (60.00)	0.00 (39.64)	0.206	0.256
Daily dietary intakes									
Energy (kcal)	1380.24 (557.85)	1525.69 (558.81)	93.22 (697.44)	0.547	1362.81 (781.51)	1568.52 (728.47)	208.81 (672.69)	0.061	0.250
Protein (g)	43.97 (29.16)	46.55 (22.14)	1.64 (22.37)	0.841	44.03 (33.87)	50.33 (25.59)	2.50 (25.22)	0.429	0.768
Total fat (g)	52.38 (22.05)	47.33 (21.83)	−7.08 (39.15)	0.111	52.14 (33.56)	62.18 (27.57)	2.48 (40.98)	0.249	0.274
Carbohydrate (g)	176.62 (87.02)	199.72 (133.69)	19.59 (115.33)	0.016 *	169.70 (129.55)	176.70 (114.66)	16.90 (136.70)	0.350	0.116
Fiber (g)	9.27 (7.04)	10.82 (7.91)	2.28 (12.53)	0.243	8.66 (8.07)	8.86 (4.91)	−0.16 (10.23)	0.785	0.308
Cholesterol (mg)	342.71 (324.60)	330.95 (262.18)	−8.98 (346.17)	0.447	325.26 (301.55)	375.15 (337.31)	54.78 (328.95)	0.252	0.110

Values expressed as median (interquartile range). ^a^ *p* values were obtained from the paired-sample Wilcoxon test. ^b^ *p* values were obtained from the Friedman rank sum test. * denotes *p* values at ≤ 0.05.

**Table 5:** Effects of SBFP and placebo on glycemic markers.

	SBFP (*n* = 38)				Placebo (*n* = 38)				*p* ^b^
	Before	After	Change	*p* ^a^	Before	After	Change	*p* ^a^	
FPG (mmol/L)	6.04 (0.86)	5.80 (0.91)	−0.14 (0.74)	0.224	5.73 (0.71)	5.79 (0.56)	0.07 (0.69)	0.155	0.045 *
PG 30 min (mmol/L)	7.16 (2.00)	7.22 (1.40)	0.17 (1.32)	0.833	6.63 (1.43)	7.17 (1.03)	0.39 (1.25)	0.123	0.413
PG 60 min (mmol/L)	7.19 (2.36)	7.63 (2.75)	0.14 (1.14)	0.080	7.40 (1.99)	7.35 (2.08)	−0.02 (1.42)	0.946	0.945
PG 120 min (mmol/L)	6.54 (1.47)	6.42 (1.69)	0.09 (0.75)	0.557	6.31 (1.69)	6.30 (1.43)	−0.06 (0.94)	0.486	0.870
2 h PG-AUC (h mmol/L)	13.69 (2.87)	14.08 (3.62)	0.40 (1.42)	0.157	13.43 (2.80)	13.54 (2.85)	0.23 (2.18)	0.636	0.871
GSP (mmol/L)	2.20 (0.20)	2.30 (0.28)	0.00 (0.20)	0.001 *	2.20 (0.20)	2.30 (0.38)	0.00 (0.39)	0.021 *	0.142

Values are presented as median (interquartile range). ^a^ *p* values obtained from the paired-sample Wilcoxon test. ^b^ *p* values obtained from mixed-model analyses. The models were adjusted for intervention, order (group), period, and individual. * denotes *p* values at ≤ 0.05.

**Table 6:** Effects of SBFP and placebo on physical examination indicators.

	SBFP (*n* = 38)				Placebo (*n* = 38)				*p* ^b^
	Before	After	Change	*p* ^a^	Before	After	Change	*p* ^a^	
SBP (mmHg)	119.50 (17.75)	113.50 (17.13)	−2.75 (12.38)	0.164	116.25 (20.13)	112.50 (17.00)	−4.50 (12.38)	0.087	0.451
DBP (mmHg)	77.25 (12.25)	73.25 (12.25)	−2.25 (6.00)	0.007 *	76.25 (13.25)	71.75 (10.88)	−3.00 (7.88)	0.006 *	0.605
BMI (kg/m^2^)	24.61 (4.81)	24.90 (4.81)	−0.04 (0.52)	0.365	25.06 (4.30)	25.02 (4.74)	−0.06 (0.46)	0.713	0.512

Values are presented as median (interquartile range). ^a^ *p* values obtained from the paired-sample Wilcoxon test. ^b^ *p* values obtained from mixed-model analyses. The models were adjusted for intervention, order (group), period, and individual. * denotes *p* values at ≤ 0.05.

**Table 7:** Effects of SBFP and placebo on glycemic markers during wash-out period.

	SBFP (group AB, *n* = 16)				Placebo (group BA, *n* = 22)				*p* ^b^
	Before	After	Change	*p* ^a^	Before	After	Change	*p* ^a^	
FPG (mmol/L)	5.66 (1.03)	5.73 (0.58)	−0.29 (0.76)	0.065	5.93 (0.56)	6.09 (0.66)	0.10 (0.59)	0.338	0.043 *
2 h PG-AUC (h mmol/L)	13.29 (2.61)	13.48 (2.41)	0.14 (1.30)	0.706	14.48 (2.99)	14.68 (3.07)	0.68 (1.15)	0.009 *	0.143
GSP (mmol/L)	2.40 (0.30)	2.25 (0.10)	−0.10 (0.13)	0.013 *	2.45 (0.38)	2.25 (0.30)	−0.20 (0.18)	<0.001 *	0.644

Values are presented as median (interquartile range).^a^ *p* values were obtained from the paired-sample Wilcoxon test; ^b^ *p* values were obtained from the two-factor repeated measurement ANOVA. * denotes *p* values at ≤ 0.05.

## Data Availability

The data that support the findings of this study have already been included in the manuscript. Raw data are available from the corresponding author upon reasonable request.
